# Frictional Behaviour of Composite Anodized Layers on Aluminium Alloys

**DOI:** 10.3390/ma13173747

**Published:** 2020-08-24

**Authors:** Radomir Atraszkiewicz, Marcin Makówka, Łukasz Kołodziejczyk, Bartłomiej Januszewicz, Jan Sucharkiewicz

**Affiliations:** 1 Institute of Materials Science and Engineering, Lodz University of Technology, Stefanowskiego 1/15, 90-924 Lodz, Poland; marcin.makowka@p.lodz.pl (M.M.); lukasz.kolodziejczyk@p.lodz.pl (Ł.K.); bartlomiej.januszewicz@p.lodz.pl (B.J.); 2 ELBIT Rp., 3th Kwietnia 21, 41-253 Czeladz, Poland; jan.sucharkiewicz@grupa-elbit.pl

**Keywords:** anodizing, friction, tribological properties

## Abstract

Three variants of the micro arc oxidation (MAO) technique have been used to treat a 2017A alloy surface. The first variant was a pure anodized layer, the second an anodized layer with SiC embedded nanoparticles and the third an anodized layer with Si_3_N_4_ nanoparticles. Tribological tests were performed for all variants, on three samples for every case. Friction coefficients and wear rates were calculated on the basis of experiments. The pure anodized layer manifested friction coefficient values within the range of 0.48 ÷ 0.52 and a wear rate in the range ~10^−15^ m^3^N^−1^m^−1^. SiC nanoparticles improved the tribological properties of the layer, as indicated by a reduction of the friction coefficient values to the range of 0.20 ÷ 0.26 with preserved very high resistance against wear (wear rate ~10^−15^ m^3^N^−1^m^−1^). Si_3_N_4_ particles embedded in anodized layer deteriorated the tribological properties, with a reduction in the resistance against fatigue and wear, intensification of friction forces and a change in the nature of friction contact behavior to more abrasive-like nature (friction coefficients ranging from 0.4 to 0.6 and wear rates ~10^−14^ m^3^N^−1^m^−1^).

## 1. Introduction

Anodizing of aluminium is mainly used to improve the tribological properties, hardness and corrosion resistance of the layered materials [1]. Anodic layers may be potentially used to protect 3D printed aluminium alloys [2,3,4,5], improve the corrosion behaviour [6] of select laser-melted aluminium alloys and protect aluminium welds [7,8].

There are many methods of producing anodized layers on aluminium alloys, from methods based on acid baths, through sol-gel technologies [9,10] to methods using high electrolytic treatment potentials, causing the presence of plasma (plasma electrolytic oxidation — PEO) or micro-arc discharges (micro arc oxidation — MAO) [11,12,13,14,15,16].

MAO methods have become an interesting solution for creating anodic layers on aluminium alloys, due to the fact that anodized layers are established with a competitive phenomenon of their dissolution by micro-discharges causing local melting of the oxide and its conversion into α-Al_2_O_3_ or γ-Al_2_O_3_ type phases, which translates directly into high operational properties of the layer. This process can be easily parameterized in terms of the proportion of individual phases and the degree of porosity [17,18].

The modification of layers with the aid of MAO methods by enriching baths with nanoparticles or covering anodized layers with non-metallic compounds is, from a tribological properties point of view, also interesting. Research in this field of work is based largely on the introduction of TiC or polytetrafluoroethylene (PTFE) nanoparticles, however, there are also solutions based on DLC (Diamond-like carbon) coatings [19,20,21,22,23,24,25].

An interesting solution, that also appeared in the field of doping anodized layers, used silicon-based compounds (SiC), but in this case the traditionally produced oxide layers were also enriched with PTFE [25]. That allowed to low friction coefficients (0.14) to be obtained while maintaining, however, low hardness of the layers (429 HV).

The solution proposed in this study is a hybrid method of composite anodized layers plasma treatment enriched with silicon-based nanoparticles (SiC, Si_3_N_4_). While in the case of silicon nitride, no beneficial effect of the nanoparticles was observed, doping with silicon carbide showed a reduction in the friction coefficient relative to pure oxide layers due to, among other causes, the reconstruction of the aluminium oxide structure.

## 2. Materials and Methods

### 2.1. Sample Preparation

The tests were carried out for set of disk samples made of the same base material aluminium alloy 2017A ASTM standard, chemical composition and main properties (presented in Table 1).

The method used for aluminium alloy treatment was micro-arc discharges (MAO) [13] with the parameters listed in Table 2.

Anodizing was carried out directly without any degreasing and activation processes and the bath temperature did not exceed 12 °C [21]. The bath was agitated during the anodizing process. Compressed air was used to mix the bath and special jet nozzles were used. The nozzles eliminate turbulent and uneven mixing with air that can cause the so-called Dead zones, where the solution is poorly mixed or mixed without mixing, which greatly improves anodizing decomposition and coating quality.

The following sets of samples were prepared for testing:

(1) oxidized to a case depth of 10 µm,
(2) oxidized to a case depth of 10 µm in a bath with 2 wt.% of SiC admixture,
(3) oxidized to a case depth of 10 µm in a bath with 2 wt.% of Si_3_N_4_ admixture.

The grain size of the SiC and Si_3_N_4_ powder added into the electrolyte medium was <250 nm.

### 2.2. Mechanical Properties

Hardness of the anodized layers was determined using nanoindentation technique on a Nano Indenter G200 system (KLA Corporation, 3 Technology Drive, Milpitas, CA 95035, USA). For nanoindentation a diamond Berkovich tip was used. The tip shape was calibrated by conducting experiments on a fused silica standard and data were analysed using the Oliver and Pharr [26] approach. Initially, hardness and modulus depth profile tests were performed using the continuous stiffness measurement mode. However, due to the high porosity of the material and abundant chipping, the obtained results were unreliable. For this reason, it was necessary to conduct the tests on the cross-section areas using the classic (basic) nanoindentation mode. The choice of the location for the measurements was carried out with built-in optical microscopy system at 800× magnification. All tests were performed under ambient conditions at maximum load of 3 mN.

### 2.3. Phase Composition Analysis of the Anodized Surface

The phase composition analysis was made by the X-Ray diffraction (XRD) method using an Empyrean diffractometer (Panalytical Empyrean, Almeo, Netherlands) working with Co Kα radiation (λ = 0.17903 nm). The X-ray source worked at accelerating voltage of 40 kV and with current value of 40 mA. A five axis X-Y-Z-Phi-Chi stage was used. For phase analysis the incident beam optics consisted of parallel beam x-ray mirror for Co radiation with 5 mm mask, anti-scatter slit 1.4 mm and Soller slits 0.04 rad was used. Scattered beam optics consisted of parallel plate collimator of divergence 0.18 deg, Soller slits 0.04 rad and proportional point Xe detector. Scan range was from 10 to 150 degrees 2Theta with 0.05 deg step size. Time per step was set to 1 s. Analysis of diffraction patterns were made with use of High Score Plus software (ver. 3.0 e, Panalytical Empyrean, Almeo, Netherlands) and ICCD PDF 4+ database. X-ray diffraction analysis was carried out for all variants of produced layers in order to check which phases are present in the layers after the anodizing process.

### 2.4. Tribological Properties

Tribological properties of the anodized substrates were investigated with the use of pin-on-disc technique, using an yttria-stabilized zirconia ball of 6.4 mm in diameter as a counterbody. For that purpose, a high temperature THT tribometer (CSM) was applied. Relative humidity (RH) and sample temperature (T) were continuously recorded during friction experiments. 

For statistics purposes, tribological tests were performed on three different samples treated in one galvanizing run. Wear track profiles after friction tests were recorded with a use of a T-1000 profilometer (Jenoptik Hommel Etamic, Jena, Germany) with at least three wear track profile measurements registered for each friction couple. The length of the measurement segment was 4.8 mm, the measurement range of the wear track depth was 80 µm and the apex (radius of the tip—2 µm) of the indenter always traversed the wear track perpendicular to the track tangent. Wear analysis for the layers against a zirconia counterpart was also carried out after friction test and specific wear rate of the layers was then evaluated according to Archard’s equation [27] on the basis of the wear track depth profiles and the total sliding distance. 

## 3. Results and Discussion

### 3.1. Mechanical Properties Results

**Figure 1:**
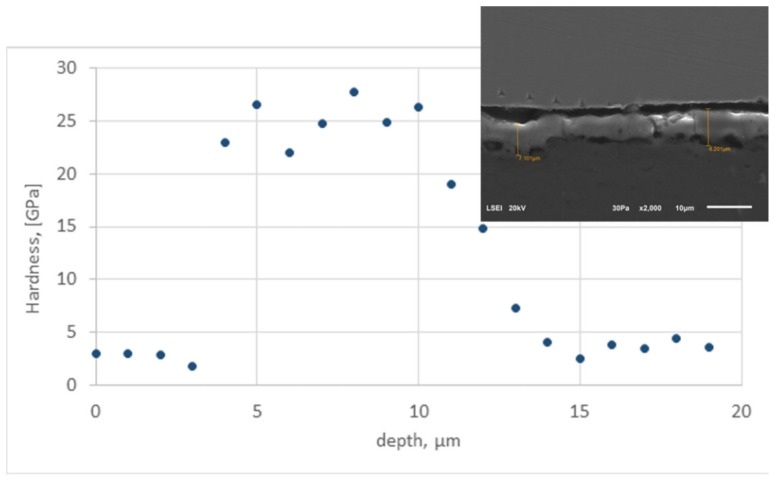
Hardness profile of 10µm thick anodized layer measured at normal load of 3 mN.

The tests were carried out on a cross-section area. Figure 1 shows the distribution of hardness results depending on the measurement location in 10 µm thick anodized layer obtained at nanoindentation load of 3 mN (indents were made across three areas: spacer made of stainless steel, anodized layer and aluminium alloy substrate, respectively). Presented hardness distributions are representative for all MAO process sets, the SiC and Si_3_N_4_ additives in the anodized layer did not significantly change the selected mechanical properties.

The hardness of the 10 µm thick anodized layer presented in Figure 1 ranged from 21 GPa to 27 GPa (the beginning of the depth profile, its first 4 points, shows the hardness values for the spacer). It is possible to observe a significant increase in hardness (more than 10 GPa) in relation to the aluminium alloy, for which the maximum obtained hardness is 5 GPa. Visible variations of the hardness results for both, layer and substrate can be attributed to porous structure.

### 3.2. Phase Composition Results

**Figure 2:**
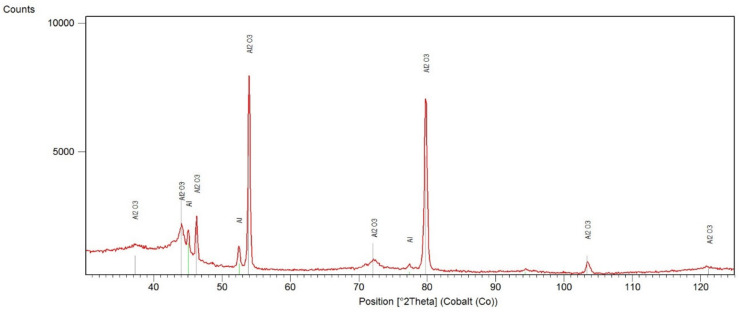
XRD pattern of anodized layer.

The analysis of XRD diffraction patterns of the pure anodized layer (Figure 2) revealed the presence of two main phases: γ-Al_2_O_3_ (ICDD reference: 00-010-0425) and A1 (ICDD reference: 04-016-4853). That is typical combination of phases after MAO process. 

**Figure 3:**
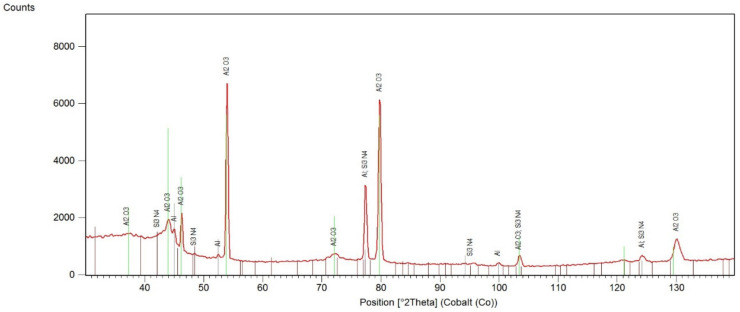
XRD pattern of the anodized layer with Si_3_N_4_ particles.

Similar results have been obtained for the layers with Si_3_N_4_ nanoparticles embedded (Figure 3). The γ-Al_2_O_3_ (ICDD reference: 00-010-0425), Al (ICDD reference: 04-016-4853) and possible traces of Si_3_N_4_ (ICDD reference: 04-007-2386) were observed.

**Figure 4:**
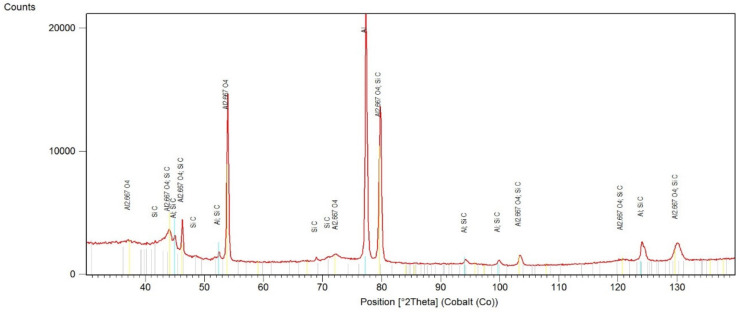
XRD pattern of the anodized layer with SiC particles.

For the layers with SiC nanoparticles embedded (Figure 4), the XRD diffraction patterns revealed presence of different phases, compared to the previous ones.

The SiC phase is observed, but instead of γ-Al_2_O_3_ phase, the η-Al_2.667_O_4_ (ICDD reference: 04-007-2615) phase has been identified. This presence of the η-Al_2.667_O_4_ phase could be explained by a reduction effect on aluminium oxide by carbon originating from the silicon carbide. This leads to formation of oxygen vacancies on the Al_2_O_3_ surface, what further provides a driving force for diffusion and crystal growth of aluminium oxide into the one dimensional structure [22].

Another hypothesis is the incomplete crystallization of alumina or due to unexpected temperature variation during the melting/crystallization of alumina during MAO process [28,29]. This is partially confirmed by the results of Ali et al. [30], who have proved that anodizing aluminium begins at lower temperatures with the formation of the η-Al_2.667_O_4_ phase, and at higher temperatures it rebuilds into Al_2_O_3_ phase.

### 3.3. Tribological Properties Results

For Al alloy with Al_2_O_3_ layer without any additives the friction couple against ZrO_2_ mean values of the coefficient of friction (COF) are in a range of 0.48 ÷ 0.52. Similar values of the coefficient of friction (–0.4) at room temperature in a friction couple with zirconia were observed in the case of a pure hardened layer on aluminium alloy produced by the micro-arc oxidation (MAO) method [31]. It is interesting that in the friction couple with Si_3_N_4_ the hardened layer produced by the MAO method (the same method as described in this paper) has a very high friction coefficient of ~0.8 [32]. COF values of the Al alloy with Al_2_O_3_ layer recorded against ZrO_2_ counterbody became greater when the Si_3_N_4_ particles were embedded in the hardened layer. One can notice that in the first ~200 m of Al_2_O_3_ + Si_3_N_4_–ZrO_2_ tribological test run the friction coefficient value decreases, and then continuously increases to the highest COF value (>0.6). The authors associate this to effect that the hardened Al_2_O_3_ with Si_3_N_4_ particles has been worn out and there was a consistent friction between pure Al alloy and ZrO_2_ counterbody. Torn out Si_3_N_4_ particles from the hardened layer and cracked fragments of the Al_2_O_3_ layer intensify the friction forces and change the nature of work in a friction contact to more abrasive-like.

As a difference, the hardened Al alloy with Al_2_O_3_ layer with embedded SiC particles exhibited the lowest COF value (0.2 ÷ 0.4). Admixture of both Si_3_N_4_ and SiC particles was expected to increase the tribological properties of Al_2_O_3_ layer. It was proved for SiC nanoparticles (COF in the range 0.2–0.4, a wear rate comparable to non-modified Al_2_O_3_) but Si_3_N_4_ caused their deterioration. The authors suspect the Si_3_N_4_ particles reduce the cohesion of the layer, therefore reducing fatigue resistance which in turn intensifies wear. Similar values of wear can be found in the literature, where a pure hardened layer produced by the MAO method was investigated in a friction couple against Si_3_N_4_ [32].

Introducing SiC particles into matrix of the η-Al_2.667_O_4_ layer (detected in X-ray analysis) leads to the reduction of COF to the value of 0.24 with preserved very high resistance (172–176) against wear (~10^−15^ m^3^⋅N^−1^⋅m^−1^). and this value is typical also for pure hardened layers produced by other methods like MAO [31].

**Figure 5:**
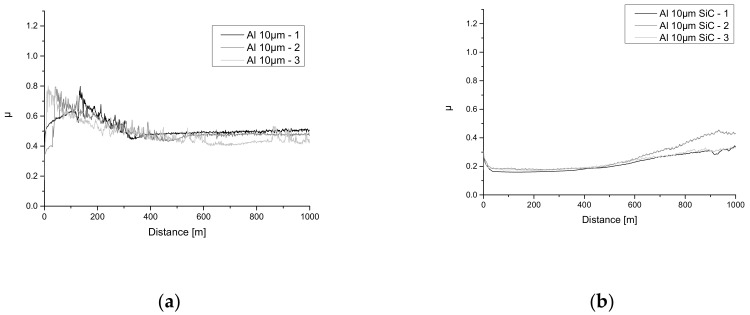

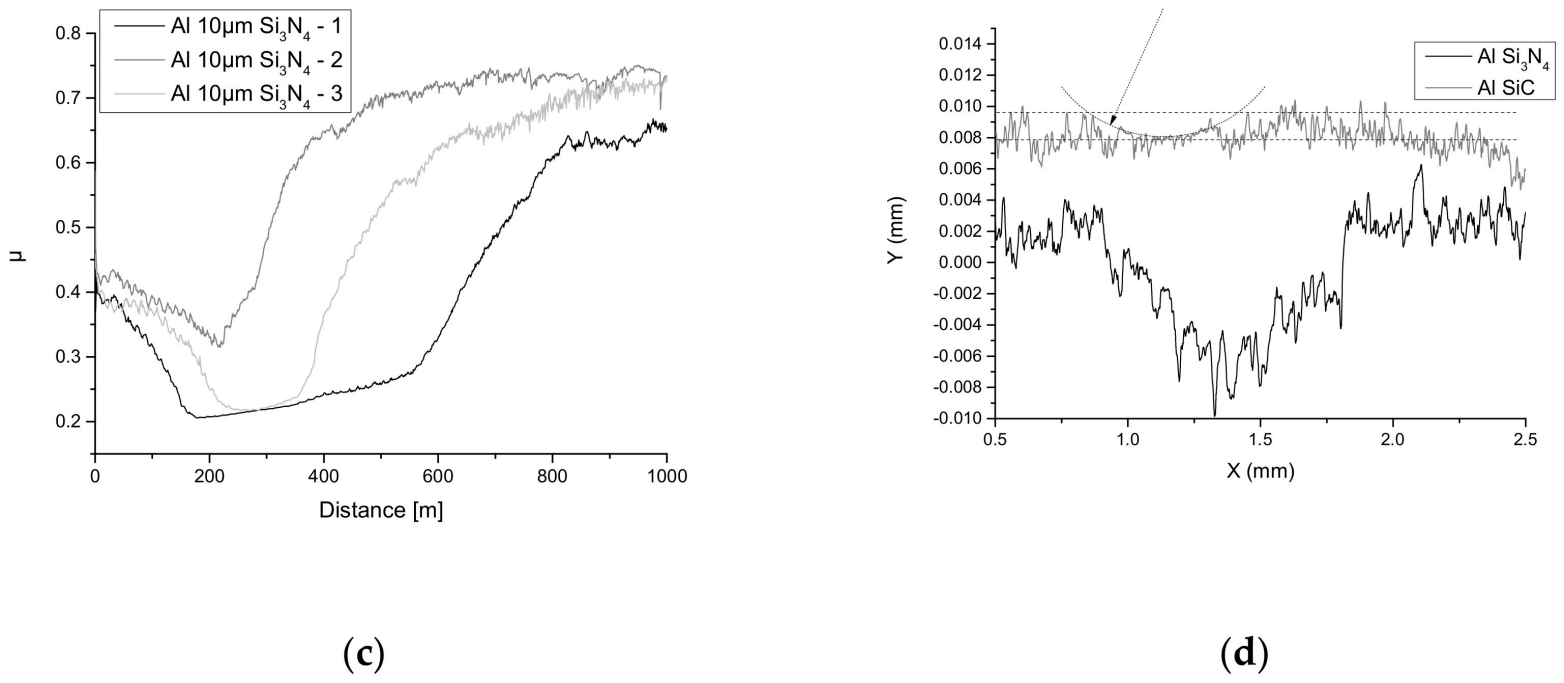
Friction coefficient evolution for pin-on-disc test against ZrO_2_ bearing ball for Al alloy with: (**a**) 10 µm thick Al_2_O_3_ layer, (**b**) 10 µm thick η-Al2.667O4 layer with embedded SiC particles and (**c**) 10 µm thick Al_2_O_3_ layer with embedded Si_3_N_4_ particles. (**d**) Example of wear track profiles after tribological tests of Al alloys with hardened layers with embedded different particles. On the upper profile (Al_2_O_3_ + Si_3_N_4_ layer) the worn area and wear profile have been marked.

Examples of COF values of the hardened Al alloys with Al_2_0_3_ layer with different additives as a function of the friction path at different pin-on-disc test conditions are shown in Figure 5, while the cumulated results of the mean COF values, the wear rates and the test conditions are collected in Table 3. Examples of wear track profiles for different Al_2_O_3_ layers after the tribological tests are shown in Figure 5. Considering that the wear was very low (for pure Al_2_O_3_ layer and layer embedded with SiC nanoparticles), it was possible to determine the order of magnitude of the k_v_ coefficient, which is sufficient for comparing the obtained results with others.

**Figure 6:**
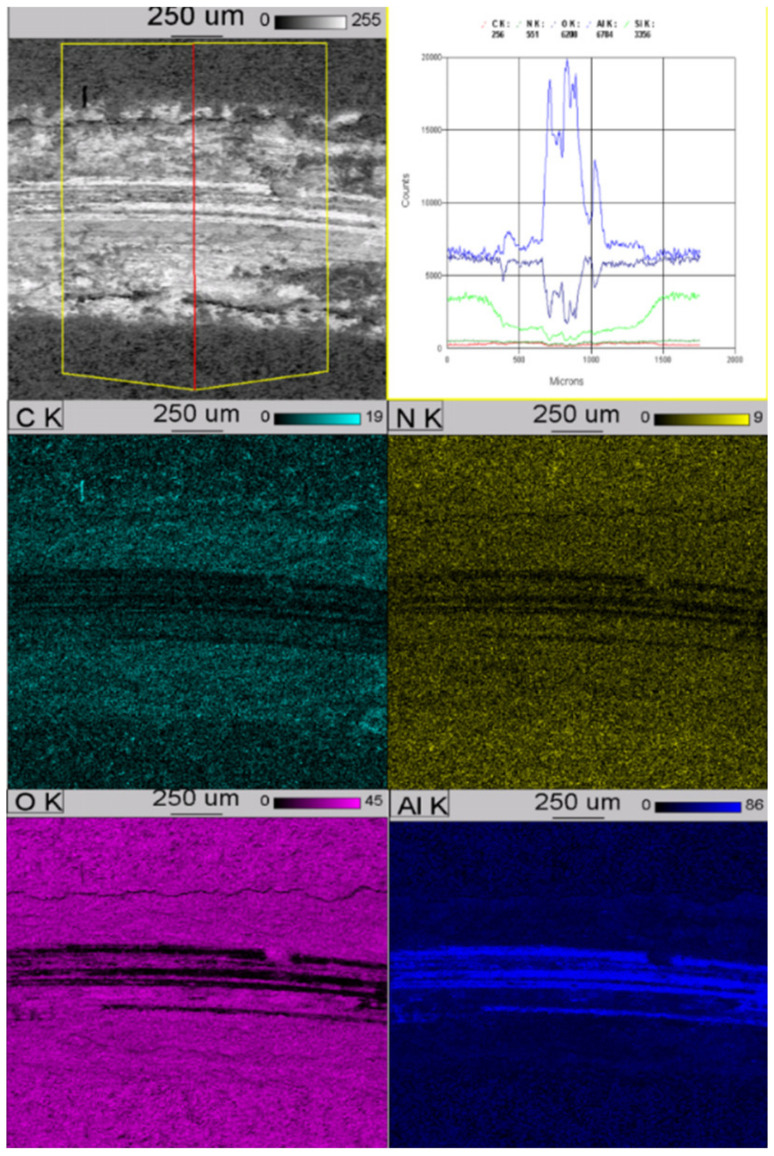
Distribution map in the area of wear of anodized layer with Si_3_N_4_ nanoparticles.

**Figure 7:**
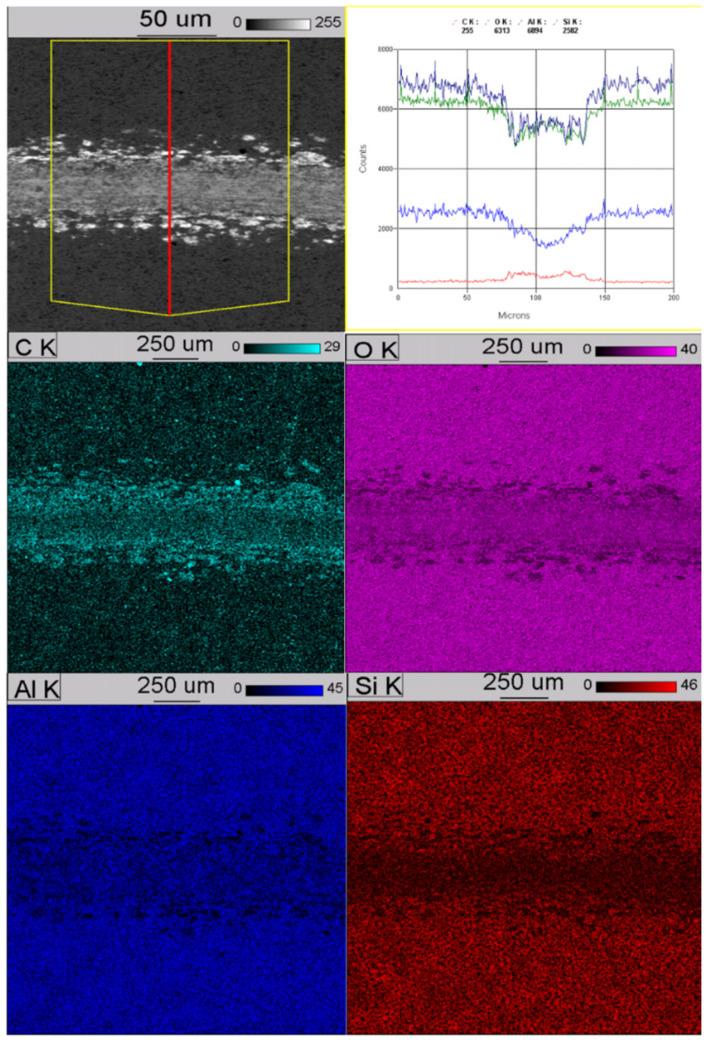
Distribution map in the area of wear of anodized layer with SiC nanoparticles.

After performing the tribological tests, microscopic tests were carried out using SEM (S-3000 microscope, Hitachi Ltd., Tokio, Japan) in the BSE mode and EDS analysis (Noran’ *Vantage* app., NORAN Instruments, Inc., Middleton, WI, USA), which was used to analyse the elemental distribution map in the area of wear. It is shown on Figure 6 and Figure 7.

Performed studies have shown that in both cases we are dealing with an even distribution of elements in the hardened layers, the exception being silicon, for which a greater concentration can be seen on the surface of the produced layers, which is the result of using Na_2_SiO_3_ + KOH solution, which remains porous during production of the layer in the final anodizing step.

## 4. Conclusions

The results of the studies presented in this work strongly indicate that the admixture of different particles (SiC or Si_3_N_4_) into the matrix of a Al_2_O_3_ hardened layer can influence the tribological properties of the resulting hardened Al alloy. Si_3_N_4_ particles embedded in the Al_2_O_3_ layer caused a deterioration of the tribological properties of that layer in the friction couple against ZrO_2_ counterbody—strongly reducing the cohesion of the layer and consequently reducing the resistance against fatigue and wear, intensifying the friction forces and changing the nature of wear in the friction contact area to more abrasive-like.

Introducing SiC particles into anodizing layer resulted instead in formation of a γ-Al_2_O_3_ phase, and the η-Al_2.667_O_4_ phase has also been identified. Presence of the η-Al_2.667_O_4_ phase could be explained by the incomplete crystallization of alumina or due to unexpected temperature variations during the melting/crystallization of alumina during the MAO process [28,30].

It seems that SiC particles present in the anodized layer after the MAO process cause the η-Al_2.667_O_4_ phase to stabilize, which means that it does not convert into γ-Al_2_O_3_ during the subsequent anodizing stage (increasing temperature in the MAO process), as is the case with clean anodized layers as well as layers with embedded Si_3_N_4_ particles. This, in turn, forms a η-Al_2.667_O_4_ phase, which is characterized by a cubic lattice, while the of γ-Al_2_O_3_ phase has a rhombohedral one [33], directly affecting the nature and intensity of wear during the tribological tests and causing an improvement of the tribological properties of the layer and reducing the COF values to the range 0.2 ÷ 0.26 with preserved very high resistance against wear (~10^−15^ m^3^⋅N^−1^⋅m^−1^).

## Figures and Tables

**Table 1:** Chemical composition and selected properties of 2017A aluminium alloy [19,20].

	Si	Fe	Cu	Mn	Mg	Cr	Zn	Ti	Other	Al
wt.(%)	0.2–0.8	0.7 max	3.5–4.5	0.4–1	0.4–0.8	0.1 max	0.25 max	0.15 max	0.15 max	balance
	Tensile strength		Yield strength		Young modulus		Hardness		Elongation	
	379 MPa		221 MPa		72.4 GPa		45 HB		22%	

**Table 2:** Anodizing parameters.

Current Density(A/dcm2)	Voltage (V)	Inductance (H)	Electrolyte	Temperature (°C)
6	500	1000	Na_2_SiO_3_ + KOH	<10

**Table 3:** Cumulative results of tribological studies.

Specimen	Load (N))	Subs. Temp. (°C)	Rel. Hum. RH (%)	Friction Radius (mm)	Friction Path(m)	Mean Value of Coef. Friction with Calculated SD	Wear Rate with Calculated SD (m^3^·N^−1^·m^−1^ × 10^−15)^
Al 10 µm–1	5	24	64	10	1000	0.48 ± 0.08	2 ± 0.6
Al 10 µm–2	5	24	64			0.51 ± 0.08	2.5 ± 0.3
Al 10 µm–3	10	27.7	63			0.52 ± 0.06	4 ± 2
Al 10 µm SiC–1	4	26	49			0.22 ± 0.06	1.4 ± 0.6
Al 10 µm SiC–2	5	21.7	60			0.26 ± 0.09	1.9 ± 0.1
Al 10 µm SiC–3	5	25.8	59.8			0.23 ± 0.05	2.5 ± 0.9
Al 10 µm Si_3_N_4_–1	4	21.5	53.4			0.4 ± 0.2	15 ± 3
Al 10 µm Si_3_N_4_–2	5	25.5	50.8			0.6 ± 0.2	20 ± 3
Al 10 µm Si_3_N_4_–3	5	26.3	48			0.5 ± 0.2	94 ± 4
